# Supplementary material for: Complexome profiling of the Chlamydomonas psb28 mutant reveals TEF5 as an early PSII assembly factor
Source: Plant Cell. 2025 Mar 17;37(6):koaf055. doi: 10.1093/plcell/koaf055 (PMC12164590; doi:10.1093/plcell/koaf055)
Supplement: koaf055_Supplementary_Data [file koaf055_supplementary_data.zip › psb28_interactive.zip!/plots/intensity/qualityPlots/peptides/DEG1C_Cre12.g498500.t1.2.html]

### Cre12.g498500.t1.2

Trivial name: DEG1C  
SeqCoverage [median]: 13.00  
Mass: 47.65 kDa  
  
 

| Sequence | MissedCleavage | Mass [Da] | wtIntensity | muIntensity | Score |
| --- | --- | --- | --- | --- | --- |
| AADLTAPSVR | 0 | 999.53 | 391.47 | 493.81 | 57.96 |
| AVSASATQR | 0 | 889.46 | 2761.71 | 3517.79 | 65.47 |
| SQTDNLFASGTAR | 0 | 1366.65 | 0.00 | 377.50 | 69.01 |
| VIGINTAIADPTGK | 0 | 1368.76 | 902.49 | 659.00 | 98.05 |
| VVGGDADKDVAVLR | 1 | 1412.76 | 1825.25 | 0.00 | 54.20 |
|  |  |  | 5880.9 | 5048.1 |  |
